# Rosiglitazone in the thawing medium improves mitochondrial function in stallion spermatozoa through regulating Akt phosphorylation and reduction of caspase 3

**DOI:** 10.1371/journal.pone.0211994

**Published:** 2019-07-05

**Authors:** José M. Ortiz-Rodriguez, Carolina Balao da Silva, Javier Masot, Eloy Redondo, Antonio Gazquez, José A. Tapia, Cruz Gil, Cristina Ortega-Ferrusola, Fernando J. Peña

**Affiliations:** 1 Laboratory of Equine Reproduction and Equine Spermatology, Veterinary Teaching Hospital, University of Extremadura, Cáceres, Spain; 2 Portalagre Polytechnic Institute, Superior Agriculture School of Elvas, Elvas, Portugal; University of PECS Medical School, HUNGARY

## Abstract

**Background:**

The population of stallion spermatozoa that survive thawing experience compromised mitochondrial functionality and accelerated senescence, among other changes. It is known that stallion spermatozoa show very active oxidative phosphorylation that may accelerate sperm senescence through increased production of reactive oxygen species. Rosiglitazone has been proven to enhance the glycolytic capability of stallion spermatozoa maintained at ambient temperature.

**Objectives:**

Thus, we hypothesized that thawed sperm may also benefit from rosiglitazone supplementation.

**Materials and methods:**

Thawed sperm were washed and resuspended in Tyrodes media, and the samples were divided and supplemented with 0 or 75 μM rosiglitazone. After one and two hours of incubation, mitochondrial functionality, Akt phosphorylation and caspase 3 activity were evaluated. Additional samples were incubated in the presence of an Akt1/2 inhibitor, compound C (an AMPK inhibitor) or GW9662 (an antagonist of the PPARγ receptor).

**Results:**

Rosiglitazone maintained Akt phosphorylation and reduced caspase 3 activation (p<0.01), both of which were prevented by incubation in the presence of the three inhibitors. Rosiglitazone also enhanced mitochondrial functionality (P<0.01).

**Conclusion:**

We provide the first evidence that the functionality of frozen stallion spermatozoa can be potentially improved after thawing through the activation of pro survival pathways, providing new clues for improving current sperm biotechnology.

## Introduction

Stallion spermatozoa can be stored in a liquid state for short periods, or it can be frozen for longer-term storage. Storing spermatozoa in a frozen state has numerous advantages; however, its widespread use is still constrained by a number of weaknesses [1]. Among them are high stallion-to-stallion variability and insufficient standardization of the freezing and thawing procedures. While cryopreservation induces mortality for an average 50% of the initial population of spermatozoa [1–4], the surviving spermatozoa are not completely functional; on the contrary, they experience accelerated senescence that requires more intense and costly mare management for insemination to compensate for this reduced lifespan. Most of the studies on sperm cryopreservation have aimed to increase the number of spermatozoa surviving the procedure, but studies aiming to improve the quality of the surviving population are scarce. Although the changes induced by cryopreservation have been extensively investigated, mostly focusing on cryopreservation induced necrosis [5–7], few studies have addressed the physiology of spermatozoa surviving freezing and thawing. There are not many studies that have tried to develop measures to increase the quality of frozen spermatozoa after thawing, with the exception of procedures to remove dead and damaged spermatozoa from the cryopreserved sample [8–10].

The population of spermatozoa surviving freezing and thawing experience changes that were recently termed spermptosis [11]. These changes basically represent acceleration of the apoptotic pathway to death that most spermatozoa undergo after ejaculation [12, 13]. In brief, osmotic shock induces membrane and mitochondrial damage [14], then the mitochondrial damage causes impairment of redox regulation, leading to lipid, protein and DNA modifications in the spermatozoa, resulting in decreased motility and viability [11, 15, 16]. Other changes recently described in relation to cryopreservation include increased intracellular Na+ and membrane depolarization due to the compromised functionality of the Na+-K+ATPase pump [17]. Akt (also called protein kinase B) plays a major role in the regulation of sperm survival. When this kinase remains phosphorylated, spermatozoa are viable, but upon dephosphorylation of Akt, pro-caspase 3 is cleaved and the spermatozoa rapidly enter a default apoptotic pathway and finally lose their ability to maintain motility [18, 19]. The cryopreserved spermatozoa show impaired mitochondrial activity due to oxidative stress and the osmotic damage that occurs during thawing [20–23]; further, cryopreserved spermatozoa present diminished mitochondrial oxygen consumption [24]. This damage compromises the capability of thawed stallion spermatozoa to produce ATP through oxidative phosphorylation [15, 25, 26]. Thus, the cryopreserved spermatozoa have lower motility and lower ATP content compromising their functionality and finally their fertilizing ability. Rosiglitazone can improve the glycolytic activity of stallion spermatozoa maintained at ambient temperature for extended periods [27]; moreover, human [28] and porcine [29] studies indicate that rosiglitazone activates pro-survival pathways in spermatozoa. In view of these facts, our hypothesis was that thawed stallion spermatozoa may also benefit from rosiglitazone supplementation.

## Materials and methods

### Animals

The ethical committee of the University of Extremadura approved the study AGL-2017-83149-R. The only manipulation of the animals was semen collection under regular veterinary practices. Six pure Spanish horses were used in this study (Table 1); the animals were fed with hay and grain, given water ad libitum and exercised daily in a horse exerciser. Ejaculates were collected using a prewarmed lubricated Missouri Model artificial vagina (Minutüb Ibérica, Tarragona, Spain) with an in line filter to eliminate the gel fraction. After collection, the semen was extended at 1:2 in INRA 96 (IMV L’Aigle France) and immediately transported to the laboratory for evaluation and processing.

**Table 1:** Summary of the stallions used in this study.

Stallion	Age	Breed	Motility % (CASA)	Morphology (% of normal spermatozoa)
1	11	PSH	89 ± 1.32	74 ± 1.33
2	12	PSH	83 ± 1.51	59 ± 1.75
3	11	PSH	71 ± 3.09	51 ± 2.1
4	8	PSH	80 ± 4.76	80 ± 1.51
5	6	PSH	87 ± 1.01	70 ± 1.26
6	13	PSH	86 ± 1.39	58 ± 1.19

Data presented as the means ± SEM

PSH Pure Spanish Horse

### Reagents and media

Hoechst 33342 (excitation: 350 nm, emission: 461 nm) (Ref: H3570); 5,5’,6,6’–tetrachloro-1,1’,3,3’tetraethylbenzymidazolyl carbocianyne iodine (JC-1) (excitation: 488 nm, emission: 530 nm, monomer form) (excitation: 561 nm, emission: 591 nm, aggregate form) (Ref: T3168); CellRox Deep Red Reagent (excitation: 644 nm, emission: 655 nm) (Ref: C10422); Cell Event Caspase-3/7 Green Detection Reagent (excitation, 502 nm, emission: 530 nm) (Ref: C10423); Annexin V Alexa Fluor 647 conjugate (excitation: 650 nm, emission: 665 nm) (Ref: A23204); and ethidium homodimer (excitation, 528 nm, emission, 617 nm) (Ref: E1169) were purchased from ThermoFisher Scientific (Molecular Probes) (Waltham, Massachusetts, USA). Anti-phospho-Akt (Ser 473) (D9E) XP Rabbit mAb (Alexa Fluor 488 conjugate was acquired from Cell Signalling Technology (Danvers, Massachusetts, USA). Rosiglitazone, dorsomorphin, GW9662 and an Akt I-II kinase inhibitor and all other reagents unless otherwise specified were purchased from Sigma-Aldrich (Madrid, Spain).

### Experimental design

**Fig 1:**
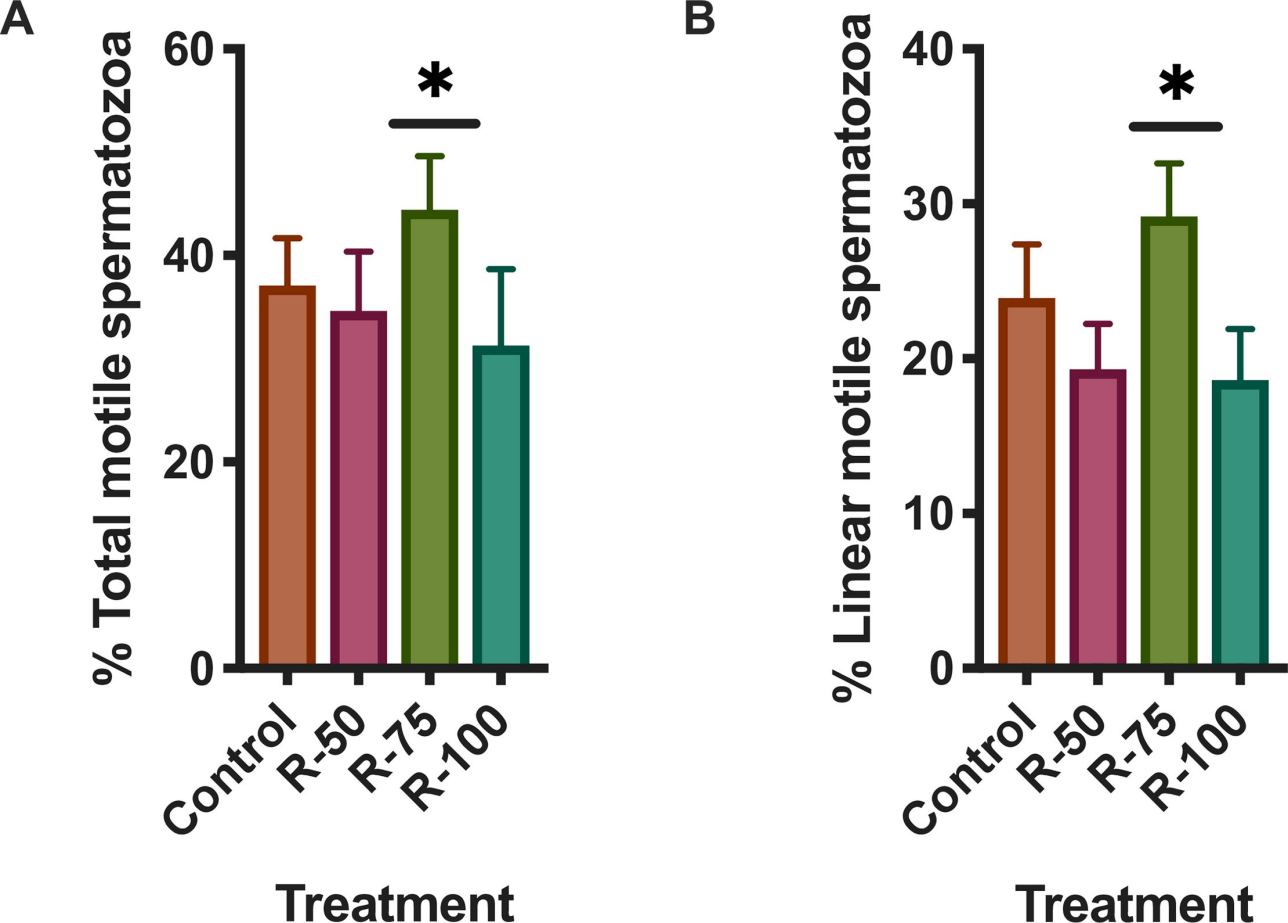
Effect of rosiglitazone added to the thawing media on stallion sperm motility. Samples were processed as described in the Materials and Methods and supplemented in the thawing media with rosiglitazone (0, 50, 75 and 100 μM) and then incubated at 37°C for two hours; then the motility was evaluated using a computer assisted system (CASA). Rosiglitazone at 75 μM increased the percentage of total (A) linearly motile (B) spermatozoa (P<0.01) (results are derived from three independent frozen ejaculates from 6 different stallions n = 18).

Frozen doses of spermatozoa (triplicate ejaculates from 6 different stallions) stored in our center were used in this study. The samples were previously obtained from stallions housed in our center as described in the section “Animals” following Institutional and European Animal care regulations (Law 6/2923 June 11^th^ and European Directive 2010/63/EU), and collected and processed following the same protocol described in previous publications of our laboratory [11, 15, 30, 31]. Straws were thawed in a water bath at 37°C for at least 30 s and were then diluted in prewarmed INRA-96 (Humeco, Huesca, Spain) extender to a final concentration of 50 × 10^6^ spermatozoa/ml. The samples were centrifuged (600 g × 10’) and resuspended in Tyrode’s media [32] to a final concentration of 50×10^6^ spermatozoa/ml. The semen was split into subsamples for control and experimental treatments and incubated in a water bath at 37°C. The doses of rosiglitazone were selected based on a previously published work [27], and incubation of the stallion spermatozoa was performed in the presence of three different concentrations (0: vehicle control DMSO 1:1000, 50, 75 and 100 μM) (Fig 1). Then, the rest of the experiments were conducted in the presence of 75 μM rosiglitazone and in the presence of specific inhibitors. Sperm functions studied included motility and kinematics, mitochondrial membrane potential, production of superoxide and live spermatozoa, caspase 3, phosphorylation of Akt and determination of the oxidation reduction potential.

### Sperm motility

Sperm motility was assessed using a Computer Assisted Sperm Analysis (CASA) system (ISAS Proiser, Valencia, Spain). Semen was loaded in a 20 μm deep Leja chamber (Leja, Amsterdam, The Netherlands) and placed on a warmed stage at 37°C. The analysis was based on an evaluation of 60 consecutive digitalized images obtained using a 10× negative phase-contrast objective (Olympus CX 41). At least three different fields were recorded to ensure that at least 300 spermatozoa were analyzed per sample. Spermatozoa with a VAP (average velocity) <15 μm/s were considered immotile, while spermatozoa with a VAP > 35 μm/s were considered motile. Spermatozoa deviating < 45% from a straight line were classified as linearly motile.

### Simultaneous detection of mitochondrial membrane potential and superoxide production

Aliquots of thawed spermatozoa were loaded with JC-1 (90 nM), CellROX deep red (2.5 μM) and Hoechst 33342 (0.25 μM) in the dark for 30 min at 22°C [33]. The samples were then run in a flow cytometer (MACSQuant VYB Miltenyi Biotech) provided with yellow laser excitation; mitochondrial membrane potential and superoxide production were investigated only in live cells using H 33342 as a viability indicator [34] and a tool to gate out non sperm events. Positive controls consisted of samples supplemented with 10 μM oligomycin to inhibit the ATP synthase a prevent incorporation of H^+^ in the mitochondrial matrix, while the negative controls were samples treated with 5 μM of the mitochondrial uncoupler CCCP.

### Measurement of oxidation-reduction potential

Oxidation-reduction potential was measured using a RedoxSYS Diagnostic system (Englewood CO, USA). This is a novel technology that measures in 4 min the static oxidation reduction potential (sORP) by measuring the potential of an electrochemical cell under static conditions, followed by measuring the capacity of oxidation reduction potential (cORP), which is the total amount of readily oxidizable molecules [35]. This technique has already been validated to determine the oxidation reduction potential of the semen and spermatozoa [36–42]. In brief, 30 μL of spermatozoa was loaded in the sample port of the pre-inserted disposable sensor, and the measurement began at the moment of loading. After 4 minutes, the static oxidative-reduction potential (sORP) is provided in millivolts (mV). ORP is calculated with the Nerst equation ORP = E^o^−RT/nF ln[Red]/[Ox], being E^o^ = standard reduction potential, R = universal gas constant, T = absolute temperature, n = number of moles of exchanged electrons, F = Faraday constant, [Red] = concentration of reduced species, [Ox] = concentration of oxidized species[41].

According to the manufacturer, sORP is measured while applying a low oxidizing current (1 nA) to the sample. After allowing 1 min and 50 s for equilibration, the reader measures twice per second over 10 s the difference in potential between the working and reference electrode in mV. Subsequently, cORP was measured by applying a linearly increasing oxidizing current until the charge rapidly changes between the working and reference electrode, indicating that all readily oxidizable molecules are fully oxidized. The time until the charge changes was used to calculate the number of electrons needed to cause charge changes and is reported in μCoulomb (μC). As controls, we used seminal plasma (rich in antioxidants)[1, 43–48], saline solution (lack of known antioxidants) and samples treated with vitamin E and menadione.

### Staining for determination of live and dead cells and caspase 3 and 7 activity

This protocol has been developed in the laboratory where the present research was conducted and has been extensively described in previous publications [15, 16, 18, 49, 50]. In brief, spermatozoa (5 × 10^6^/mL) in 1 mL of PBS were stained with CellEvent 2 μM and 0.5 μM Hoechst 33342 and incubated for 25’ in the dark at 22°C. Then, 0.3 μM of Eth-1 was added to each sample and after incubation for 5 additional minutes the samples were immediately evaluated in a flow cytometer (Cytoflex flow cytometer, Beckman Coulter). CellEvent staining was validated using western blotting as described in [50]. Positive controls were obtained by incubating stallion spermatozoa at 37°C for 3 hours in the presence of three known inductors of apoptosis [51–55], staurosporine 10 μM, thapsigargin 50 μM and betulinic acid 50 μM.

### Simultaneous assessment of caspase 3 activity and phosphatidylserine (PS) translocation

Spermptotic changes were detected in spermatozoa with the use of Annexin V 674 conjugate (Molecular Probes, Leiden Holland), which detects the translocation of phosphatidylserine (PS) from the inner to the outer leaflet of the plasma membrane and is associated with membrane changes related to sperm processing and with the CellEvent Caspase 3/7 Green Detection Reagent. This consists of a four amino acid peptide (DEVD) conjugated to a nucleic acid binding dye. The activation of caspase 3 and caspase 7 proteins enables them to cleave the caspase 3/7 recognition sequence that is encoded in the DEVD peptide. Cleavage of the recognition sequence and binding of the DNA by the reagent labels apoptotic cells[56]. Both stains were combined in a multiparametric test and evaluated by FC[57]. A final concentration of 5 × 10 ^6^ spermatozoa/ml was obtained by adding 10 μL of diluted spermatozoa to 990 μL of Annexin Binding Buffer. Then, the samples were loaded with Hoechst 33342 (0.3 μM) and CellEvent (2 μM) and incubated at room temperature for 15 minutes. Next, the samples were washed by a short centrifugation spin for 12”and suspended in 200 μl of Annexin binding-buffer (solution in 10 mM HEPES, 140 mM NaCl, 2.5 mM CaCl_2_, pH 7.4). To 200 μL of sample per assay, 5 μL of Annexin V was added. After 15 minutes of incubation in the dark at room temperature, 400 μL of 1 × Annexin binding-buffer was added before reading it in the flow cytometer (Cytoflex flow cytometer, Beckman Coulter).

### Detection of phosphorylated AKT (Ser ^473^) in stallion spermatozoa

Samples were washed in PBS and fixed with 2% paraformaldehyde in PBS for 10 minutes at 4°C; after fixation, the cells were washed (centrifuged at 473 ×g for 8 minutes at room temperature) twice with PBS and once with PBS-1% BSA, and permeabilized for 30 min in 0.1% saponin in PBS-1% BSA. Then, the samples were stained with 2 μL/ml of phospho-AKT Alexa fluor 488 conjugate (cat n° 4071, Cell Signalling Technology) and incubated in PBS-1% BSA for 30 min in the dark at room temperature. Samples were then washed again in PBS and analyzed in the flow cytometer (Cytoflex flow cytometer (Beckman Coulter). This flow cytometry protocol has been previously validated in our laboratory [18].

### Flow cytometry

Flow cytometry analyses were conducted using a Cytoflex flow cytometer (Beckman Coulter) equipped with violet (405 nm), blue (488 nm) and red lasers (635 nm) and a MACSQuant VYB (Miltenyi Biotech) flow cytometer equipped with yellow (561 nm), violet (405 nm), and blue lasers (488 nm) as sources of excitation. The quadrants or regions used to quantify the frequency of each sperm subpopulation depended on the particular assay. Forward and sideways light scatter were recorded for a total of 50,000 events per sample. Gating the spermatozoa population after Hoechst 33342 staining eliminated nonsperm events. The instruments were calibrated daily using specific calibration beads provided by the manufacturers. A compensation overlap was performed before each experiment. Files were exported as FCS files and analyzed using FlowjoV 10.4.1 Software (Ashland, OR, USA). Unstained, single-stained, and Fluorescence Minus One (FMO) controls were used to determine compensations and positive and negative events, as well as to set regions of interest as described in previous publications from our laboratory [18, 58, 59].

### Computational flow cytometry (t-SNE analysis)

Flow cytometry data are usually analyzed using a series of 2D plots and manual gating; however, the increase in the number of parameters measured increased the number of 2D plots to display for every marker combination. For example, a combination of four colors requires 30 2D plots. To overcome these problems, computational methods to automatically identify populations in multidimensional flow cytometry data have been developed [60]. Using Flowjo v 10.5.3 software (Ashland, OR, USA) compensated data of each multiparametric assay as described in the material and methods, the data were exported as FCS files from the flow cytometer and loaded into Flowjo for computational analysis. Data were downsampled, concatenated and single cell events analyzed. Flow cytometry data were analyzed using non-linear dimensional reduction techniques (t-SNE). This technique identifies clusters within multidimensional data without losing single cell resolution [61, 62], allowing for automatic gating of cells. Within the t-SNE maps generated, heat maps were applied to identify the expression of specific markers.

### Statistical analysis

Frozen semen samples were obtained from 6 different stallions. All experiments were repeated at least three times with independent samples (three separate ejaculates from each of the donor stallions). The normality of the data was assessed using the Kolmogorov-Smirnoff test. Since the data were normally distributed, paired t tests and one-way ANOVA followed by Dunnett’s multiple comparisons test were performed using GraphPad Prism version 7.00 for Mac, GraphPad Software, La Jolla California USA, www.graphpad.com. Overton cumulative histogram subtraction was also performed [63] to determine positivity in selected cytometry analysis; in brief, this method determines the percent of the events that are considered to have positive florescence for the selected parameter by subtracting out the florescence of the control. Differences were considered significant when P < 0.05 and are indicated as the following: * P<0.05 and ** P<0.01. Results are displayed as the means ± SEM.

## Results

### Rosiglitazone in the thawing media improves sperm motility

Post thaw incubation with rosiglitazone (75 μM) showed significant improvements in motility after two hours of incubation (P<0.05, one way ANOVA, n = 18). Both the percentages of total motile spermatozoa (36.0 ± 5.0 in controls vs 45.0±5.2% in samples supplemented with rosiglitazone 75 μM) and linear motile spermatozoa showed significant improvements (P<0.05, one way ANOVA n = 18) with the treatment (Fig 1A and 1B). Other concentrations of rosiglitazone tested had no effect.

### Rosiglitazone enhances mitochondrial function in thawed stallion spermatozoa

**Fig 2:**
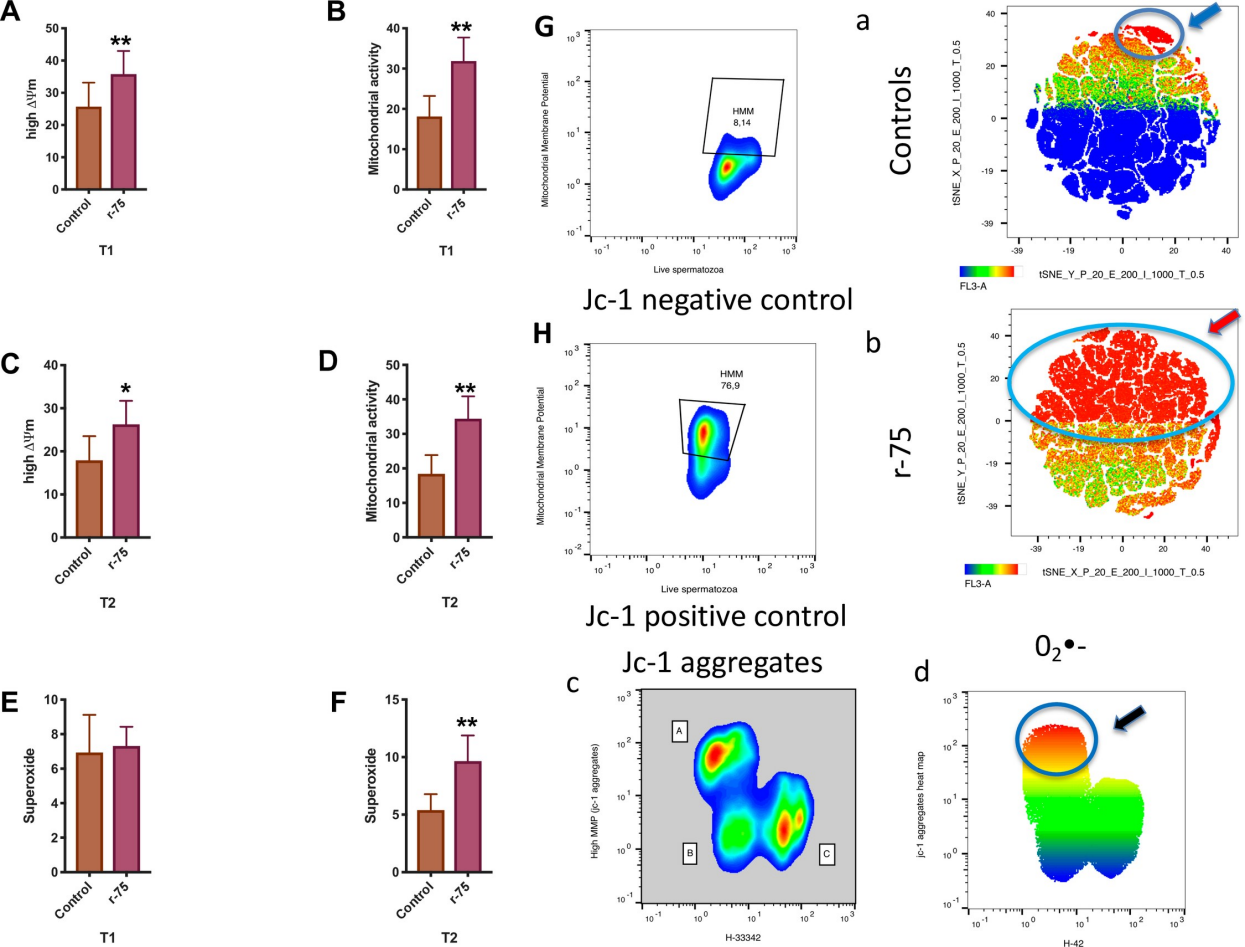
Effects of rosiglitazone added to the thawing media on mitochondrial function of stallion spermatozoa after thawing. Frozen stallion semen was thawed and processed as described in the Materials and Methods. Split samples were supplemented with rosiglitazone (0 and 75 μM) and mitochondrial functionality was investigated after 1 and 2 hours of incubation. A and C, percentage of spermatozoa showing orange fluorescence after JC-1 staining, B and D, mitochondrial functionality expressed as the mean fluorescence intensity in the PE channel indicative of JC-1 aggregates (high mitochondrial potential), E and F production of superoxide after 1 and 2 hours of incubation at 37°C. The results are presented as the means ± SEM. * P<0.05, ** P<0.01. In a and b, the t-SNE map after computational analysis is shown; in the t-SNE map, each point represents individual spermatozoa in the sample, and the heat map applied to the t-SNE map shows increased PE fluorescence (jc-1 aggregates) in the rosiglitazone treated samples. Circles identify the populations of spermatozoa showing high ΔΨm. In c a representative 2D plot after JC-1/H33342 is presented, A live spermatozoa with high ΔΨm, B live spermatozoa, C dead spermatozoa. In d the same plot is presented but a heat map overlay of the APC channel (production of superoxide) is shown over the 2D plot depicted in c, maximum production of superoxide is present in live sperm showing high ΔΨm (orange events in the plot), and this population is also circled (black arrow). Controls for the JC-1 are presented; G negative controls that are samples treated with the mitochondrial uncoupler CCCP 5 μM. Positive controls are presented in H; they are samples treated with oligomycin 10 μM to inhibit the passage of H^+^ to the mitochondrial matrix (results are derived from three independent frozen ejaculates from 6 different stallions n = 18).

Mitochondrial impairment is a hallmark of thawed stallion spermatozoa [11, 22, 64, 65] and is also considered an early event in spermptosis [11]. To determine if rosiglitazone is able to improve mitochondrial function, thawed stallion spermatozoa were incubated in the presence of rosiglitazone (75 μM), and after one and two hours of incubation the mitochondrial function of the surviving spermatozoa was investigated using JC-1. Since the production of the superoxide anion (0_2_•^-^) is a byproduct of oxidative phosphorylation in the mitochondria [66, 67], the production of 0_2_•^-^ was concurrently investigated. Rosiglitazone significantly increased (P<0.05 and P<0.001, two tails paired t test, n = 18, after 1 and two hours of incubation, respectively) the mitochondrial potential of the surviving spermatozoa at both timepoints examined (Fig 2A–2D). Increased mitochondrial activity estimated as an increased presence of JC-1 aggregates [56] also occurred (P<0.05 two tails paired t test n = 18) without concomitant increases in the production of 0_2_•^-^ after 1 hour of incubation (Fig 2E), but there was a significant increase (P<0.01, two tails paired t test, n = 18) in 0_2_•^-^ in the supplemented samples after 2 hours of incubation at 38°C (Fig 2F). When the analysis was performed on a cell by cell basis of the whole sperm population, the heat map generated after the t-SNE analysis showed evident changes indicating that rosiglitazone increased mitochondrial activity (estimated as the number of JC-1 aggregates) compared with controls (Fig 2A and 2B) in the whole sperm population, although the changes varied in degree. Additionally, to identify the major source of 0_2_•^-^, a heat map was generated for superimposing the APC channel (CellRox deep red) over the JC-1/H33342 2D plot (Fig 2C), showing that the major production of 0_2_•^-^ occurred in the more active mitochondria (Fig 2D, blue circle and black arrow).

### Effect of rosiglitazone on the oxidation reduction potential (sORP)

**Fig 3:**
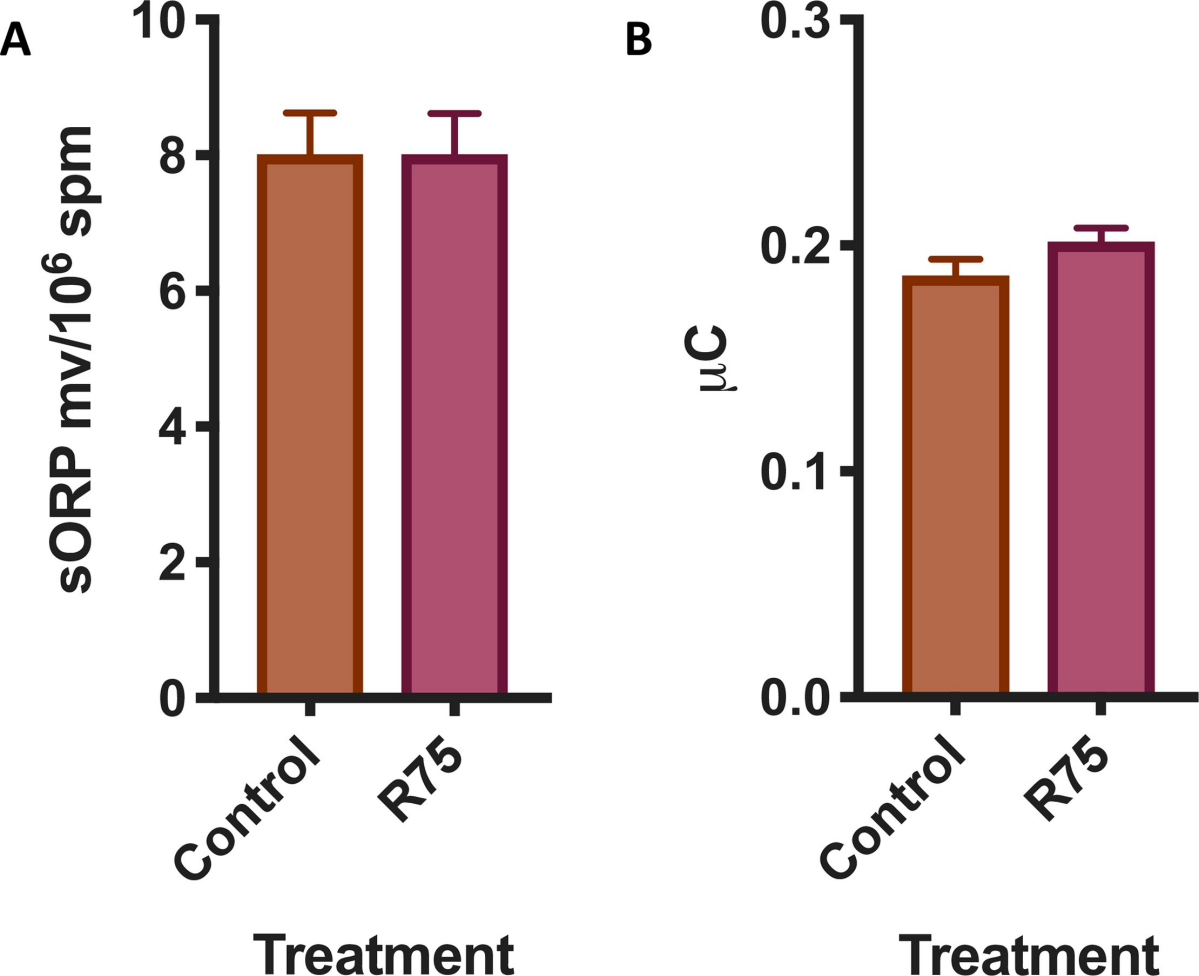
Effect of rosiglitazone added to the thawing media on sORP (mV/10^6^ sperm) (A) that is the integrated measure of the existing balance between oxidants and reductants and (B) antioxidant capacity reserve cORP (μC) (results are derived from three independent frozen ejaculates from 6 different stallions n = 18).

To determine if the increased production of superoxide is just caused by intense mitochondrial activity [66] or is a sign of oxidative stress, the oxidation-reduction status of the samples was investigated. No changes were observed in the static oxidation reduction potential (sORP) or in the total antioxidant capacity in the supplemented samples (Fig 3) (n.s., two tails paired t test, n = 18).

### Rosiglitazone reduces caspase 3 activation without changes in phosphatidylserine transposition

**Fig 4:**
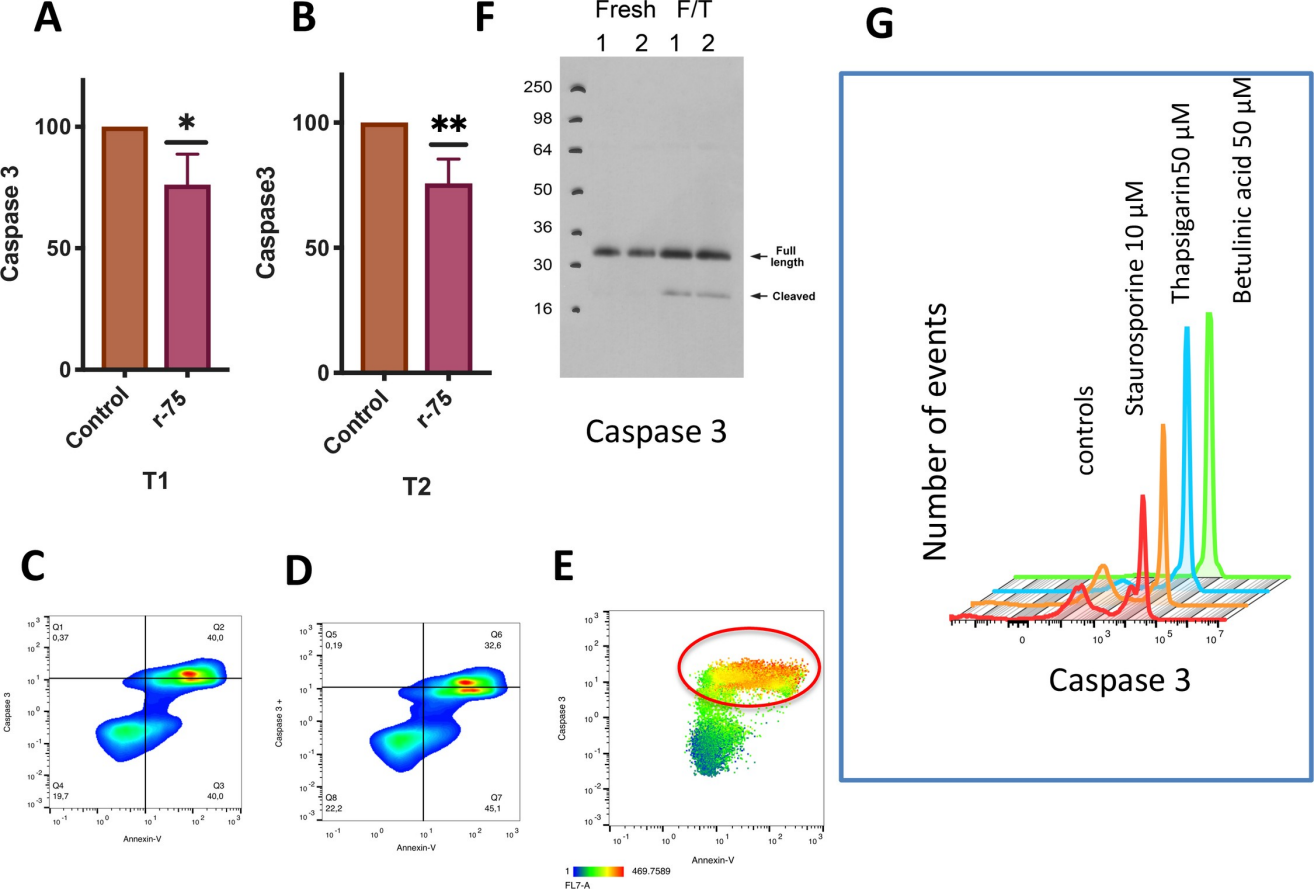
Changes in caspase 3 and phosphatidylserine (PS) transposition after rosiglitazone supplementation of the thawing media. Commercial frozen doses of stallion sperm were thawed and processed as described in the Material and Methods. Split samples were incubated in the presence of rosiglitazone 0 and 75 μM and caspase 3 activity was determined by flow cytometry. Data represent percent changes with respect the controls after 1 hour (A) and two hours (B) of incubation and are expressed as the means ± SEM, observed (* P<0.05, results are derived from three independent frozen ejaculates from 6 different stallions n = 18). F) Western blot (WB) controls for caspase 3 using frozen and thawed stallion spermatozoa as positive controls; semen was processed and analyzed as described in reference 50. (G) Further controls were obtained after incubating stallion spermatozoa at 37°C for 3 hours in the presence of three known inductors of apoptosis, staurosporine 10 μM, thapsigargin 50 μM and betulinic acid 50 μM. In C and D, representative cytograms of the simultaneous detection of active caspase 3 and PS transposition are presented where Q2 and Q3 represent events positive both for caspase 3 and Annexin-V. Q2 represents events with higher caspase 3 expression. No significant changes were detected. In E a heat map showing the intensity of Annexin-V staining demonstrates that PS is preferentially expressed in caspase 3 positive cells.

**Fig 5:**
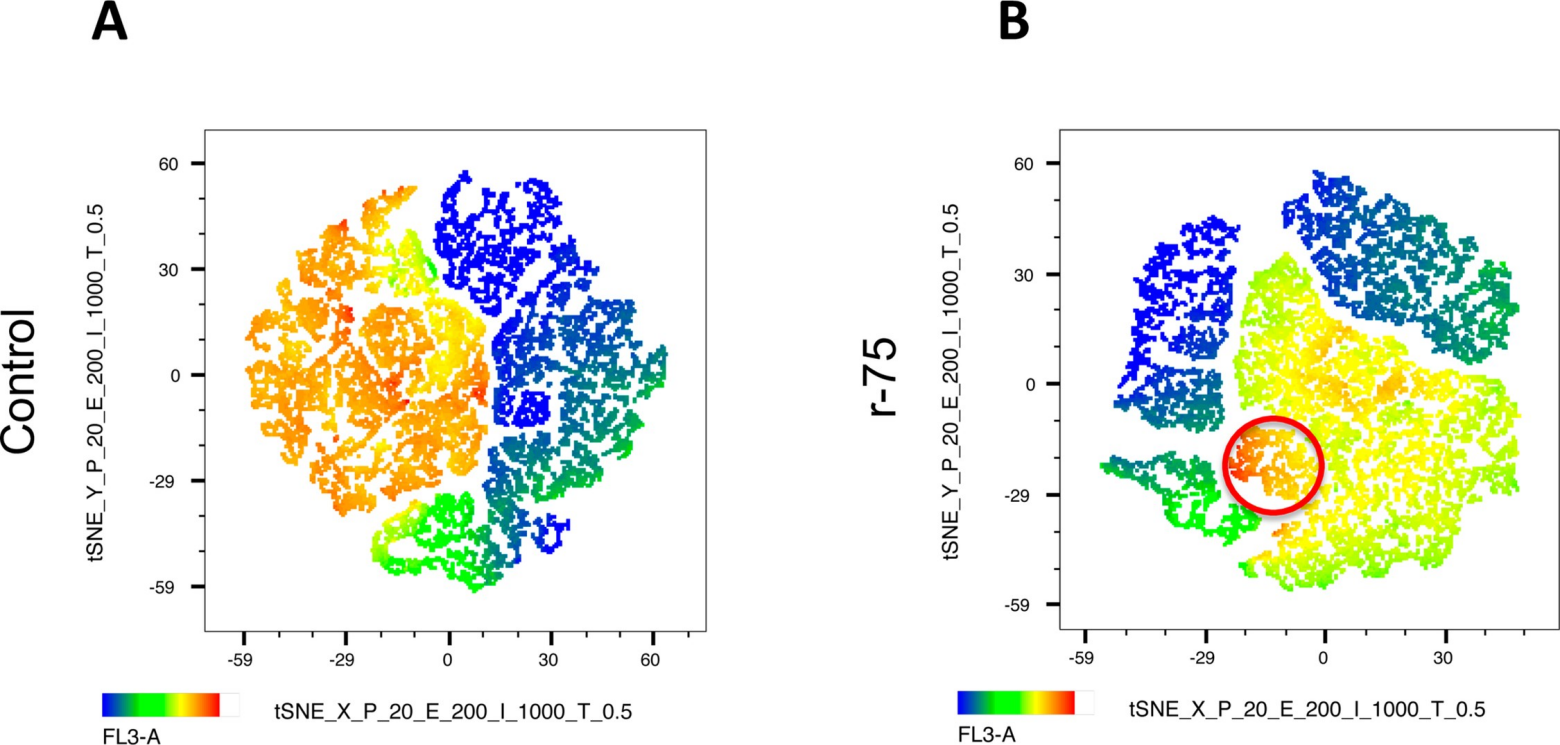
Computational cytometry analysis (t-SNE) graphics. Heat maps are presented showing the effect of 75 μM rosiglitazone supplementation on caspase 3 activity in thawed stallion spermatozoa. A, Control samples, each point represents an individual spermatozoa, and as seen in the heat map, almost half of the population shows high caspase 3 expression (orange color). B, Samples supplemented with 75 μM rosiglitazone, and as seen in the heat map, caspase 3 expression is reduced with only a small population with high caspase 3 (red circle) (results are derived from three independent frozen ejaculates from 6 different stallions n = 18).

Since it has been reported that caspase activation triggers sperm senescence [18], we studied the effect of rosiglitazone on caspase 3 activation and phosphatidylserine (PS) transposition; when thawing media was supplemented with 75 μM rosiglitazone, a significant decrease of >25% in respect to the initial values in controls after 1 and 2 hours of incubation occurred (two tails paired t test, n = 18, P<0.05, P<0.01, respectively) (Fig 4A and 4B). Study of the t-SNE map also showed a decrease in caspase 3 activation (Fig 5A and 5B). No changes induced by rosiglitazone were observed in PS (Fig 4C and 4D). The combined 2D dot plot and heat map of the Annexin-V fluorescence intensity revealed that most of the caspase 3 positive spermatozoa were also Annexin-V positive (Fig 4E, red circle).

### Rosiglitazone phosphorylates Akt and increases the percentage of live non apoptotic spermatozoa

**Fig 6:**
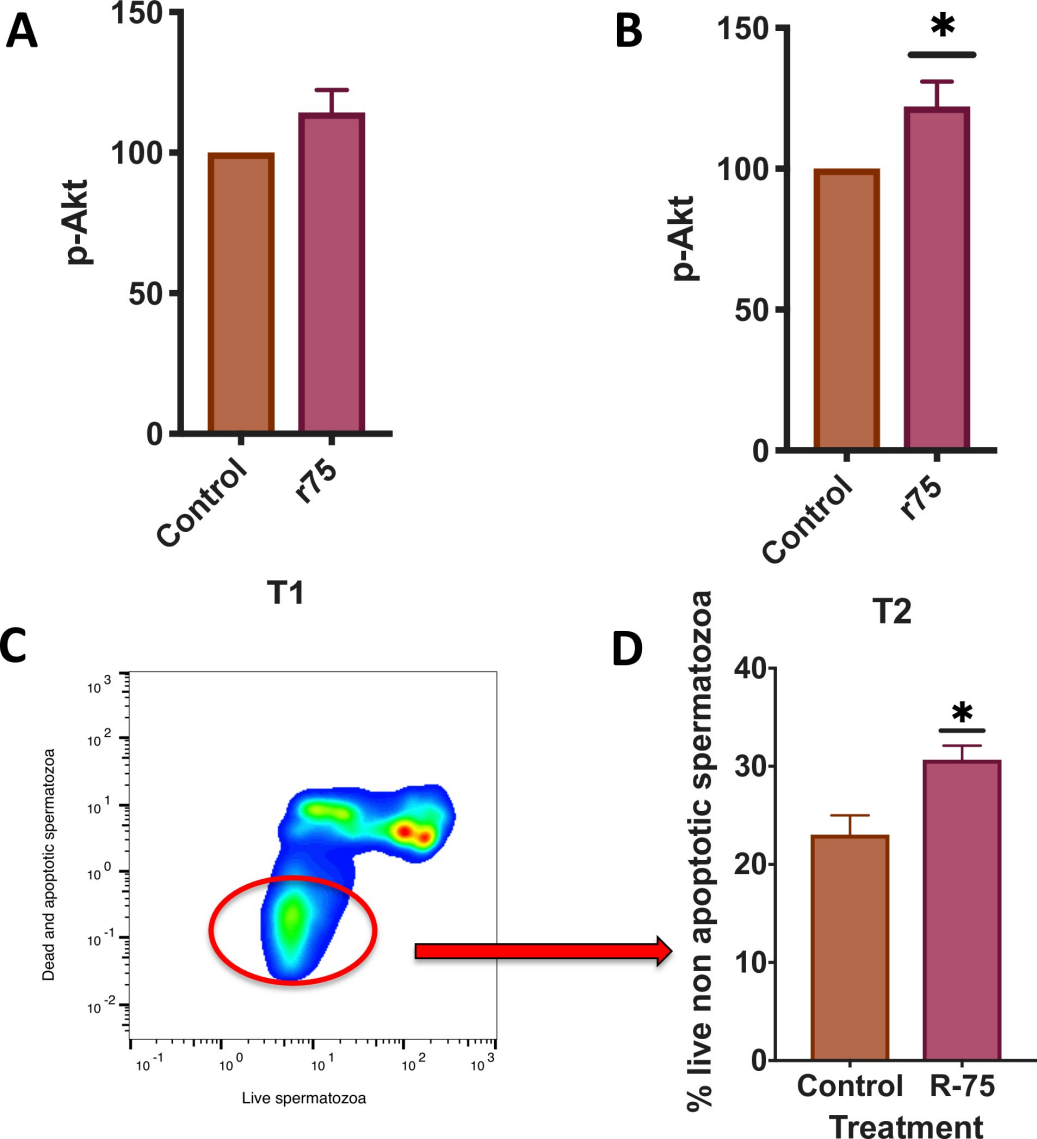
Effect of rosiglitazone on Akt phosphorylation (Ser ^473^) on stallion spermatozoa and on the percentage of live non apoptotic spermatozoa (caspase 3 negative). Commercial frozen doses of stallion sperm were thawed and processed as described in the Materials and Methods. Split samples were incubated in the presence of rosiglitazone 0 and 75 μM and Akt phosphorylation was measured after 1 (A) and 2 hours (B) of incubation at 37°C. Data represent percent changes with respect to the controls and are expressed as the means ± SEM * P<0.05. In C, a representative cytogram showing the identification of live non apoptotic spermatozoa is shown. Live spermatozoa are identified by the red circle. In D the effect of the incubation of stallion spermatozoa in the presence of rosiglitazone 75 μM is presented. Data represent percent changes with respect to controls and are expressed as the means ± SEM * P<0.05 (results are derived from three independent frozen ejaculates from 6 different stallions n = 18).

**Fig 7:**
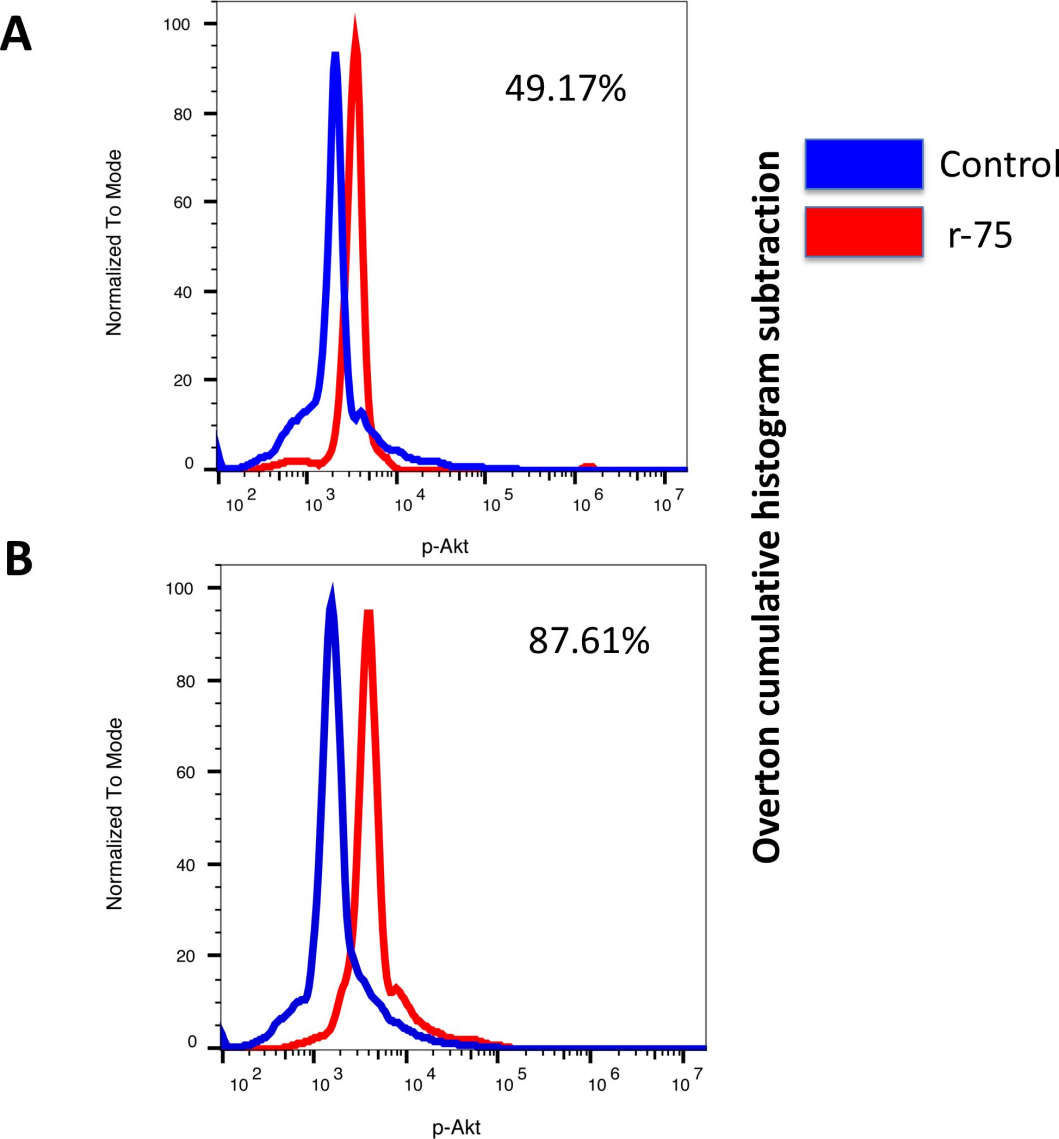
Representative overlay cytograms of the p-Akt assay after 1 hour (A) and 2 hours of incubation (B). To calculate the expression of the different germ cell markers we used the population comparison analysis available in FlowJo, version 10.4.1 (TreeStar, OR, USA). This analysis uses the Overton cumulative histogram subtraction algorithm (Overton, 1988) and overlaps histograms of the control (isotype control) and sample, allowing for subtraction of the control to calculate the percentage of positive cells in the sample (percentage of cells showing increased expression with respect to the controls) (results are derived from three independent frozen ejaculates from 6 different stallions n = 18).

Previous findings from our laboratory linked the dephosphorylation of Akt to the activation of caspase 3 in ejaculated stallion spermatozoa [18]. We hypothesized that rosiglitazone may be linked to Akt phosphorylation. Incubation of thawed stallion spermatozoa in the presence of rosiglitazone maintained phosphorylated Akt after two hours of incubation at 37°C in comparison with untreated controls (two tails paired t test, P<0.05, n = 18) (Figs 6 and 7). Furthermore, samples incubated in the presence of 75 μM rosiglitazone showed an increased percentage of live non apoptotic spermatozoa, 23.0 ± 1.9 in controls vs 35.0± 1.4% in 75 μM rosiglitazone supplemented samples (P<0.05, two tails paired t test, n = 18).

### Inhibition of Akt, PPARγ and AMPK abolished the reduction of caspase 3 activation induced by rosiglitazone

**Fig 8:**
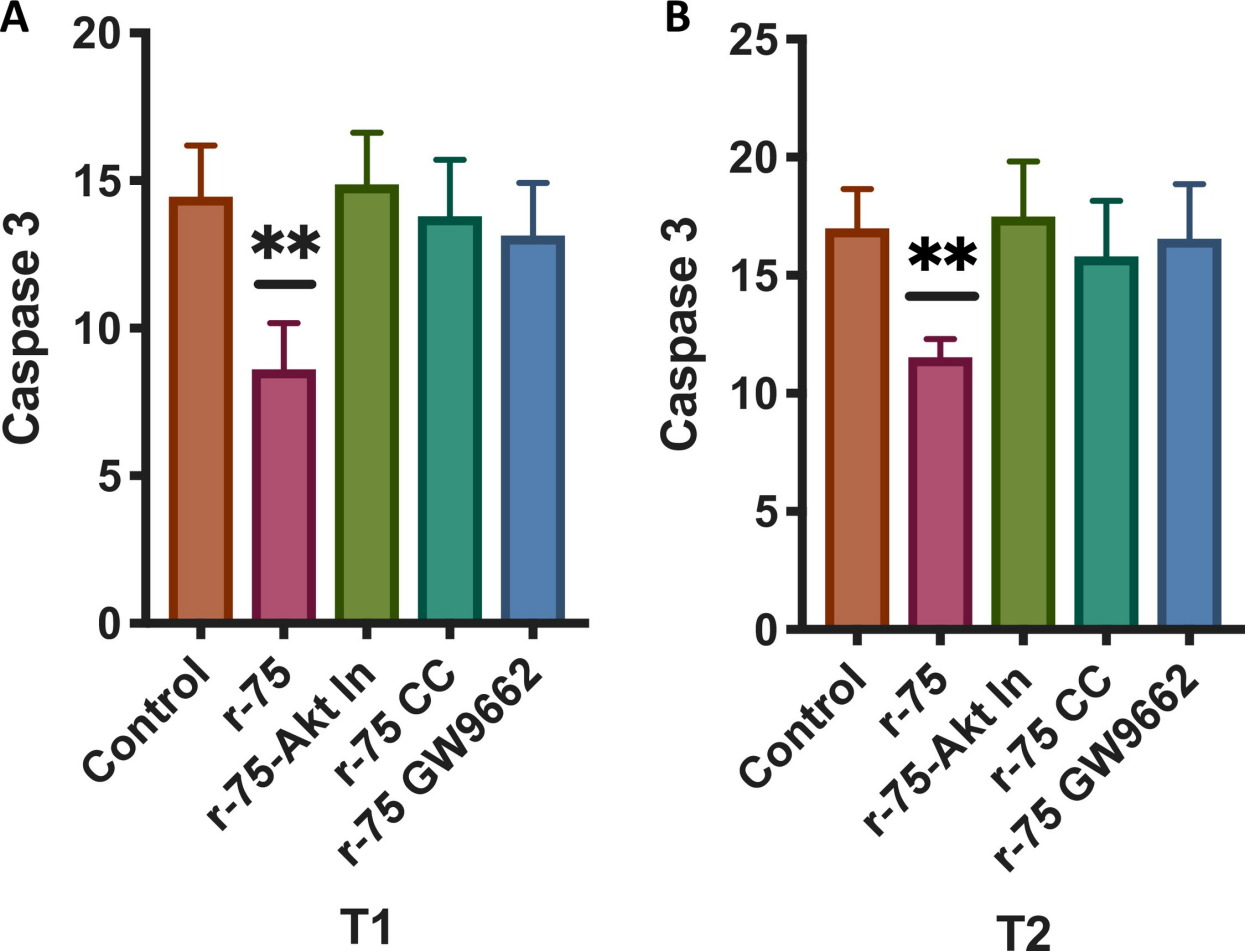
Effects of the Akt1/2 kinase inhibitor, dorsomorphin (AMPK inhibitor) and GW9662 (PPAR γ inhibitor) on caspase 3 inhibition after rosiglitazone treatment. Thawed semen doses were processed as described in the Materials and Methods and were incubated in the presence of rosiglitazone (0 and 75 μM) or rosiglitazone 75 μM plus an Akt kinase inhibitor 30 μM, rosiglitazone 75 μM plus GW9662 10 μM or rosiglitazone 75 μM plus dorsomorphin 100 μM. After 1 and 2 hours of incubation caspase 3 activity was determined using flow cytometry. The results are presented as the means ± SEM. * P<0.05 A) changes after 1 hour of incubation, B) Changes after 2 hours of incubation (results are derived from three independent frozen ejaculates from 6 different stallions n = 18).

The effects of rosiglitazone can be mediated by the PPARγ receptor and/or by the phosphorylation of the AMPK[68–70]. In both cases, Akt can be phosphorylated [29]. To determine if the effects observed from supplementing with rosiglitazone could be reverted by inhibiting PPARγ, pAMPK and AKT phosphorylation, samples were incubated in the presence of an Akt1/2 inhibitor (30 μM)[18], GW9662 (inhibitor of PPARγ, 10 μM)[27] and dorsomorphin (an inhibitor of AMPK, 100μM)[27] and then incubated in the presence of rosiglitazone 75 μM. As seen in the previous experiment, rosiglitazone reduced caspase 3 activation (one way ANOVA, n = 18, P<0.01); however, when the samples were incubated in the presence of rosiglitazone and the three inhibitors, at one and two hours of incubation the reduced caspase 3 activation induced by the rosiglitazone was no longer present (Fig 8A and 8B).

## Discussion

In this study, we aimed to determine whether the quality of frozen stallion spermatozoa can be improved after thawing. Traditional approaches to improve sperm survival after freezing and thawing have focused on the improvement of extenders, sperm selection prefreezing or post thawing, and freezing and thawing rates [8, 71–77]. Few studies have focused on the development of methods to improve sperm quality after the thawing phase; moreover, few studies have addressed the biology of thawed spermatozoa. Our results show that the functionality of thawed stallion spermatozoa can be improved through the activation of pro-survival pathways; in particular, its mitochondrial function can be significantly improved using this strategy.

Recent studies point to stallion spermatozoa as highly dependent on intracellular thiols for their proper functionality [15, 30, 78] and highly dependent on oxidative phosphorylation in the mitochondria as the main source of ATP for motility, but they act mainly in maintenance of membrane functionality [21, 65, 67, 79, 80]. These facts have important implications for the selection of more fertile spermatozoa [67] and sperm conservation [20, 79–81]. The thawed spermatozoa are characterized by compromised mitochondrial function [11, 17] and an unstable redox status, leading rapidly to oxidative stress [1, 17, 48]. We aimed to induce metabolic flexibility to improve the functionality of thawed spermatozoa, a strategy that has proven successful in the conservation of stallion spermatozoa kept at ambient temperature for long periods [27]. Moreover, we studied the potential mechanisms behind this improvement. The PPAR γ agonist rosiglitazone induced clear improvements in mitochondrial function and reduced caspase 3 activity and these effects were also linked to increased phosphorylation of Akt. Previous reports indicate the importance of Akt phosphorylation in sperm function [18, 19, 82, 83] and recently a link between PPARγ agonists and Akt phosphorylation in human [28] and pig spermatozoa [29] has been reported. Moreover, strategies to maintain Akt phosphorylation in spermatozoa have proven to be successful in human sperm cryopreservation [82, 84].

The approach described in our work allowed us to maintain p-Akt (the phosphorylated form) in thawed spermatozoa. Moreover, the use of specific Akt inhibitors provided further support to the proposed relationship between p-Akt and proper sperm function, as reported for human [19, 28] and equine spermatozoa [18, 85]. It may be of practical importance to underscore the fact that phosphorylated Akt can be maintained in thawed sperm through the use of rosiglitazone; this fact may indicate that after thawing spermatozoa, in spite of the dramatic osmotic stress occurring during the procedure, the spermatozoa may maintain mechanisms to regulate their lifespan. This is an interesting finding, since it opens a new approach to develop strategies to improve the quality of frozen spermatozoa after thawing. The balance between survival or death pathways activation may depend on the capability to regulate redox homeostasis [16]. In different cellular models, Akt regulates mitochondrial function, and this regulation is not necessarily dependent on transcriptional activity [86], supporting the proposed mechanism described here of enhanced mitochondrial function after PPARγ agonist treatment in spermatozoa. Further supporting this hypothesis, treatment of stallion spermatozoa with Akt inhibitors prevented improvements after rosiglitazone treatment, as did inhibition of PPARγ and AMPK, although a negative effect of the inhibitor in the absence of rosiglitazone cannot be excluded. However, the inhibitor treatments indicated that in stallions most of the effect of rosiglitazone may be related to AMPK activation, since this effect has been previously reported in stallion spermatozoa maintained in the liquid state [27]; in addition, the PPARγ inhibitor was less efficient at reverting rosiglitazone’s effects. More interestingly, rosiglitazone enhanced mitochondrial function while maintaining redox homeostasis; although increased superoxide production was observed after two hours, the oxidation reduction potential sORP did not change, suggesting that, as previously reported [24], increased production of superoxide may be an indicator of intense mitochondrial activity. However, although superoxide production is somewhat mitochondrial specific, it can also be produced by NADPH oxidases in the sperm head. Additionally, there is some evidence that mitochondrial ribosomes in the spermatozoa are both transcriptionally and translationally active [87], so the possibility that rosiglitazone may be acting through translational pathways warrants further investigation.

Cryopreservation depletes the thiols in spermatozoa, causing an unstable redox status that rapidly evolves to redox deregulation [88]. This situation induces caspase 3 activation and sperm death. The results reported here show that this form of sperm death can be delayed. In fact, we observed that supplemented samples showed a higher percentage of live non-apoptotic spermatozoa after two hours of incubation (Fig 6D). The positive outcome of rosiglitazone supplementation reported here can be attributed to the activation of metabolic flexibility. In this way, spermatozoa may be more effective at using glycolysis [27] and β oxidation of fatty acids [89] for energy production. Additionally, as revealed in our experiment, this pathway improves the efficiency of mitochondrial function. Mitochondrial function is considered a hallmark of functional spermatozoa [90–92], and more fertile stallion samples show more active mitochondria [67]; these reports support the concept that the quality and fertilizing ability of thawed samples can be modulated after thawing.

In conclusion, thawed stallion spermatozoa can be improved post thaw through mechanisms that maintain Akt in the phosphorylated state, which is a process that may involve AMPK and PPARγ activation. Moreover, these findings may have practical applications to improve the quality of thawed samples independently of the initial freezing protocol.
